# Theoretical and Experimental Design of Heavy Metal-Mopping Magnetic Nanoparticles

**DOI:** 10.1021/acsami.0c17759

**Published:** 2021-01-03

**Authors:** Elia Roma, Pietro Corsi, Max Willinger, Nikolaus Simon Leitner, Ronald Zirbs, Erik Reimhult, Barbara Capone, Tecla Gasperi

**Affiliations:** † Dipartimento di Scienze, Universitá degli Studi Roma Tre, Via della Vasca Navale 84, 00146 Roma, Italy; ‡ Department of Material Sciences and Process Engineering, University of Natural Resources and Life Sciences, Vienna, Peter-Jordan-Strasse 82, A-1190 Vienna, Austria

**Keywords:** biocompatible nanoparticles, polymers, poly(2-oxazoline)s, thermoresponsive, heavy metal, core−shell nanoparticles, magnetic

## Abstract

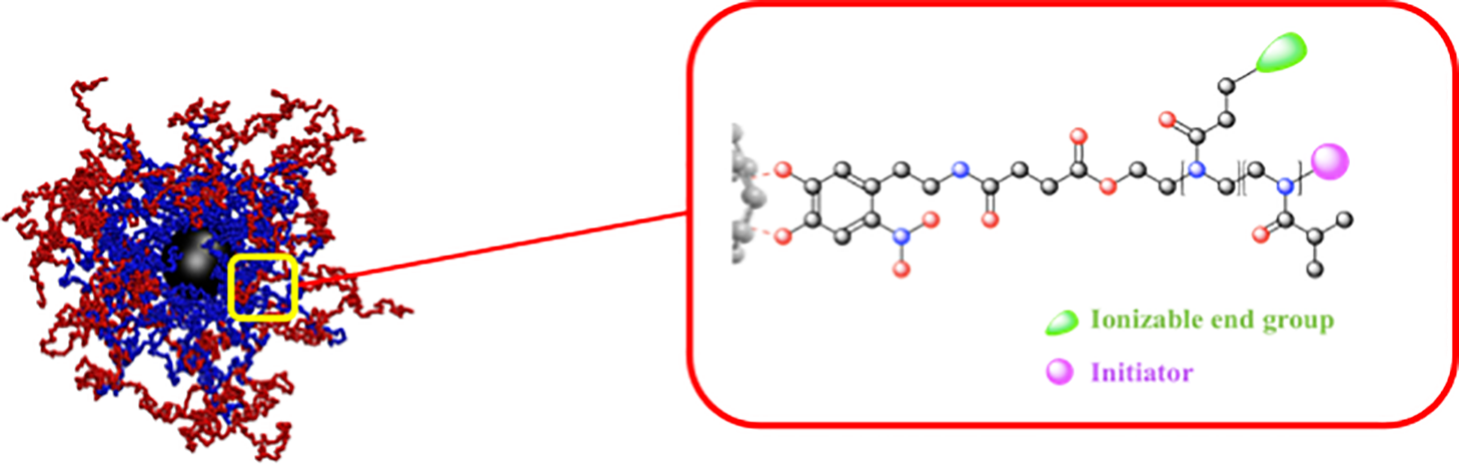

Herein, we show a comprehensive experimental, theoretical, and computational study aimed at designing macromolecules able to adsorb a cargo at the nanoscale. Specifically, we focus on the adsorption properties of star diblock copolymers, *i.e.*, macromolecules made by a number *f* of *H-T* diblock copolymer arms tethered on a central core; the *H* monomeric heads, which are closer to the tethering point, are attractive toward a specific target, while the *T* monomeric tails are neutral to the cargo. Experimentally, we exploited the adaptability of poly(2-oxazoline)s (POxs) to realize block copolymer-coated nanoparticles with a proper functionalization able to interact with heavy metals and show or exhibit a thermoresponsive behavior in aqueous solution. We here present the synthesis and analysis of the properties of a high molecular mass block copolymer featured by (i) a polar side chain, capable of exploiting electrostatic and hydrophilic interaction with a predetermined cargo, and (ii) a thermoresponsive scaffold, able to change the interaction with the media by tuning the temperature. Afterward, the obtained polymers were grafted onto iron oxide nanoparticles and the thermoresponsive properties were investigated. Through isothermal titration calorimetry, we then analyzed the adsorption properties of the synthesized superparamagnetic nanoparticles for heavy metal ions in aqueous solution. Additionally, we use a combination of scaling theories and simulations to link equilibrium properties of the system to a prediction of the loading properties as a function of size ratio and effective interactions between the considered species. The comparison between experimental results on adsorption and theoretical prediction validates the whole design process.

## Introduction

In recent years, synthesis and investigations of polymer properties have focused on the design of macromolecules, able to perform predetermined tasks and sensitive to external stimuli. Specifically, the ability of the adsorbing macromolecules to react to chemo-physical changes, such as temperature, pH gradients, and magnetic fields, renders them as a promising and tunable material for controlled adsorption and release at the nanoscale.^1−3^ Polymeric macromolecules have shown to be an extremely promising system in the adsorption/release framework. The adsorption process arises from the interplay between the enthalpic attraction amid the cargo and the macromolecule and the entropic repulsion due to excluded volume interactions between the monomers belonging to the polymeric macromolecule as well as between monomers and the colloidal particles.^4^ The possibility for a macromolecule to satisfy multiple weak bonds with specific regions on target surfaces has been demonstrated to enhance the selectivity in the adsorption process when compared to macromolecules that are able to satisfy fewer and stronger interactions.^5−8^ Being made of hundreds to thousands of monomeric units, each one of which can interact selectively with a particular cargo in solution, polymeric macromolecules are optimal candidates as a precise tunable smart nano-adsorber. Among functionalizable smart nanomaterials, magnetic nanoparticles with responsive solubility have risen to a prominent position due to their remarkable properties and widespread applications, spanning from drug delivery to extractions of pollutants from solutions; their biocompatibility and low toxicity open the path toward their application in both biomedical and biotechnological sectors,^9,10^ while their unique magnetic properties are interesting in the field of material science.^11,12^ Recently, the synthesis of highly monodisperse iron oxide cores grafted with polymer brushes, bearing specific functional end groups,^13,14^ paved the way for the design of shell architectures with detailed control over interactions between nanoparticles and either biomolecules or inorganic compounds. Colloidally stable small superparamagnetic nanoparticles (SPMNPs, <15 nm) are efficiently dispersed but difficult to catch by means of a magnetic separator.^15−17^ This major drawback can be solved by coating the SPMNPs with a tunable polymer shell. The possible polymeric coatings,^12,18,19^ the poly(2-alkyl/aryloxazoline)s (PAOx), gained significant interest due to their tunable properties, biocompatibility, stealth behavior, and thermosensitivity.^17,20−24^ The living cationic ring-opening polymerization (CROP) of 2-oxazoline provides easy access to a wide variety of well-defined PAOx, with controlled end-group functionality, and variation of the side chain substituent,^25−29^ which are mainly introduced during the 2-oxazoline monomer synthesis yielding functional PAOx. Among 2-alkyl-2-oxazoline monomers, 2-isopropyl-2-oxazoline (iPrOx) have raised particular attention ascribable to the similarity of the resulting polymer (PiPrOx) with poly(*N*-isopropylacrylamide) (PNIPAAm), its structural isomer. Specifically, PiPrOx exhibits a lower critical solution temperature (LCST) in water around 38 °C, which is just above body temperature,^30^ and higher than that of PNIPAAm (LCST = 32 °C). This makes PiPrOx a good candidate for the design of thermoresponsive polyoxazoline-based polymer that can undergo a phase transition in pure water, *i.e.*, from a dispersible state at low temperature to a collapse state at different conditions.^31−34^ This work focuses on the design, synthesis, and characterization of a new macromolecule that has been developed to perform a pre-determined action in aqueous solution. In particular, we compare theoretical predictions to experimental realizations and characterizations with the aim of highlighting the main features that render a spherical core–shell polymeric nanoparticle an efficient adsorbing system. Specifically, we prepared the colloidally stable superparamagnetic nanoparticles (SPMNPs) grafted with diblock copolymer, which are able to

- change the interaction with the media by tuning the temperature, a crucial property that allows for a reversible aggregation of the SPMNPs into larger clusters that respond strongly to magnetic fields and can be easily filtered away.^15,16^
- interact and adsorb heavy metals in aqueous solution; the SPMNP process mimics the chelating effects of the most common drugs (*i.e.*, deferoxamine and ethylenediaminetetraacetic acid (EDTA))^8^ currently employed for the treatment of heavy metal body intoxication.

Moreover, we report an in-depth study of the thermoresponsive behavior of novel, densely grafted polymeric nanocomposites as well as an initial interpretation of their ability to adsorb heavy metal ions in aqueous solution.

Furthermore, the experimental observations on the adsorption process are supported by a theoretical and computational analysis. To gather the main elements leading to the adsorption process in the experimental conditions, the adsorbing macromolecule is represented theoretically as a soft colloidal particle, while heavy metals (cargos) are represented as hard colloidal particles. The paradigm of soft colloidal particles is star polymers, *e.g.*, macromolecular assemblies made by a number *f* of polymeric arms, each of *N* monomers, that are grafted to a central core.^35,36^ Properties of binary mixtures of star polymers and colloidal particles have been widely investigated over the past decades, especially when no interaction is set between the two species.^37−40^ In this case, for a fixed concentration of the two species, the properties of the binary mixtures are mainly influenced by the size ratio *q* = *R*_g_/*r*_c_, between the average radius of gyration of the star polymer *R*_g_ and the colloid radius *r*_c_.^4,41,42^ When an interaction ε is set between the two species, the binary mixture phase diagram is determined by the (*q*, ε) combination.^4^ There is a critical (*q*, ε) combination that leads to the development of an effective attractive interaction between the soft adsorbing colloid and the hard adsorbed one. In this paper, we show that, when the cargo is much smaller than the adsorbing macromolecule, adsorption takes place, and a typical shrinkage of the adsorbing macromolecule is predicted as a function of the maximum loading. Moreover, we predict both theoretically and computationally the same order of magnitude of adsorbed macromolecules as what is estimated experimentally for all of the cases.

In summary, with this work, we realize experimentally, assess computationally, and support theoretically a macromolecular system that is able to adsorb heavy metal ions in solution. Our findings sets a starting point for a further and more in-depth evaluation of the optimal conditions for adsorption and hence selectivity in adsorption.

## Results and Discussion

### Experiments

**Scheme 1:**
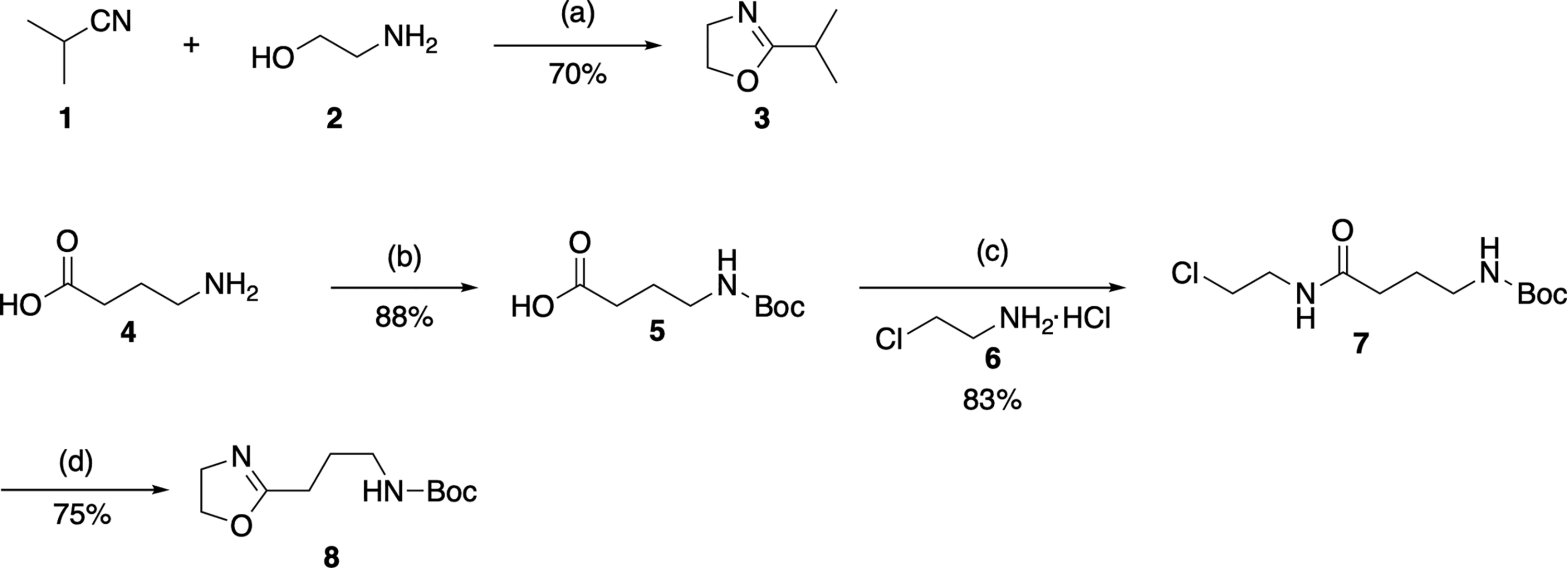
Synthesis of 2-Alkyl-2-oxazoline Monomers **3** and **8** Reagents and conditions: (a) Zinc acetate (Zn(OAc)_2_), 130 °C, overnight; (b) aqueous solution of NaOH (5.5 M), Boc_2_O, rt, overnight; (c) *O*-(benzotriazol-1-yl)-*N*,*N*,*N*′,*N*′-tetramethyluronium tetrafluoroborate (TBTU), **6**, Et_3_N, 0 °C to rt, overnight; (d) NaOH in MeOH saturated solution, 12 h, rt.

The preparation of monomers, 2-isopropyloxazoline (iPropOx, **3**) and the novel *tert*-butyl(3-(4,5-dihydrooxazol-2-yl)propyl)carbamate (AmOx, **8**) were successfully accomplished by modifying and optimizing the reaction conditions of various procedures described in the literature.^43,44^ Shortly, iPropOx (**3**) was formed by refluxing isobutyronitrile and ethanolamine with Zn(OAc)_2_ as a catalyst (Scheme 1). For the novel monomer, AmOx (**8**), a three-step synthetic procedure was followed. Initially, the amino Boc protection was performed on *γ*-aminobutyric acid (**4**) to isolate the product **5**. Subsequently, the carboxylic function was modified with chloroethylamine using a coupling reagent *O*-(benzotriazol-1-yl)-*N*,*N*,*N*′,*N*′-tetramethyluronium tetrafluoroborate (TBTU), affording the last intermediate **7**, which was further cyclized, according to the stability of the amino protecting group, in basic conditions, to achieve the desired product as white solid **8** (Scheme 1).

**Scheme 2:**
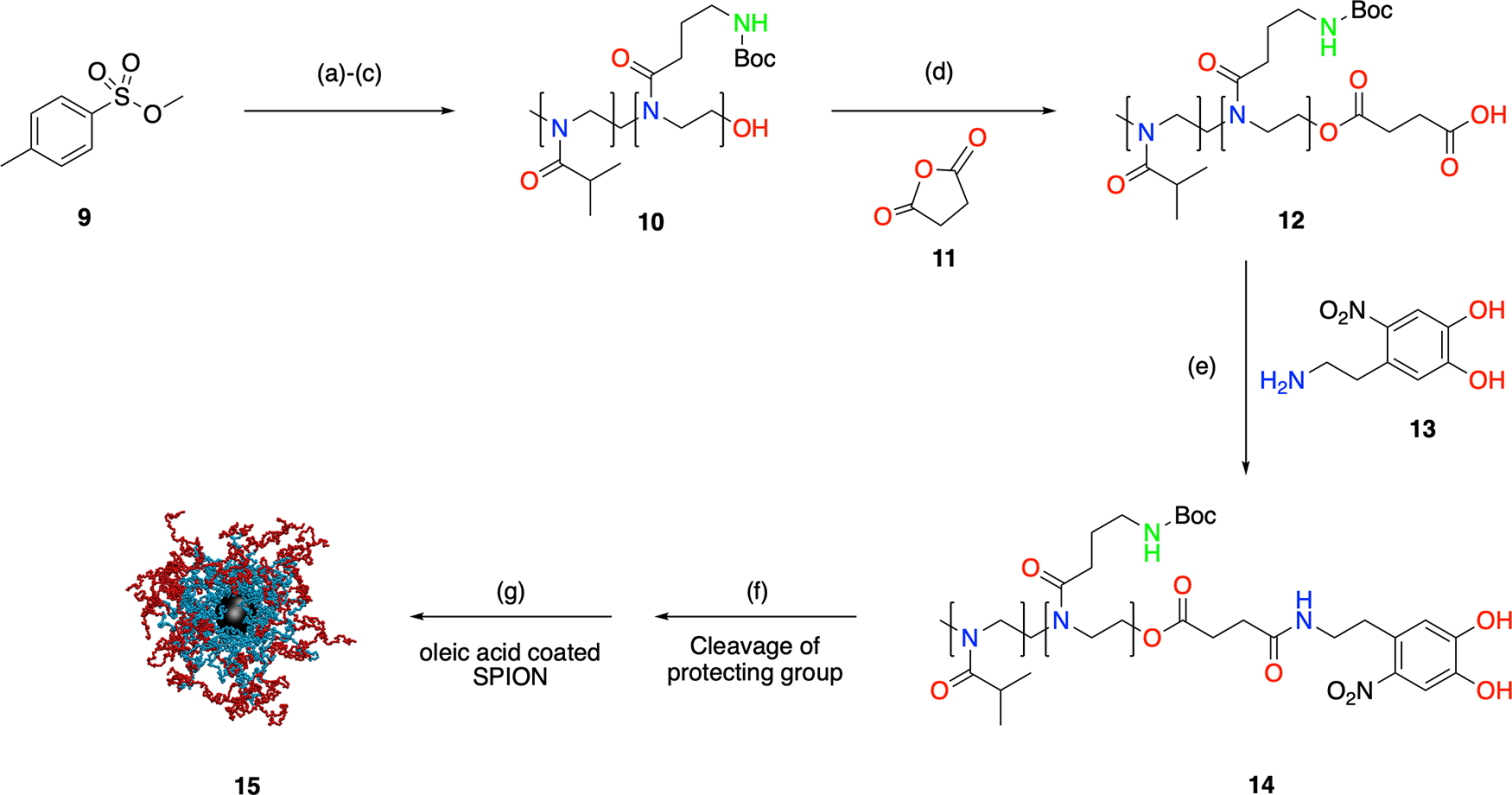
Synthetic Pathway to Block Copolymer-Modified SPMNPs Reagents and conditions: (a) iPropOx, DMA, 100 °C, 7 h; (b) AmOx, DMA, 100 °C, 12 h; (c) NaOH (2 M), rt, 12 h (d) **11**, Et_3_N, toluene, reflux, 24 h; (e) **13**, (1-cyano-2-ethoxy-2-oxoethylidenaminooxy)dimethylamino morpholino carbenium hexafluorophosphate (COMU); *N*,*N*-diisopropylethylamine (DIPEA), 0 °C to rt, 3 days; (f) trifluoroacetic acid (TFA):DCM (1:1), overnight, rt; (g) superparamagnetic iron oxide nanoparticle (SPION), DMA, 20 °C, under ultrasonication, 24 h.

The polyoxazoline block copolymers (**14**) were synthesized using methyl *p*-toluenesulfonate (MeTos) as an initiator. In this case, the good agreement between [M]_0_/[I]_0_ and the obtained degree of polymerization as well as the small polydispersity index indicates a living (stoichiometric) cationic polymerization. Specifically, the synthesis was performed by sequential polymerization of 2-isopropyloxazoline (iPropOx, **3**) and the second monomer, 2-aminobocoxazoline (AmOx, **8**), in DMA at 100 °C. Quenching with water generated the hydroxy-terminated polymers. This was further modified by direct esterification with succinic anhydride. Finally, the amidation with nitrodopamine furnished nitrodopamine terminated block copolymers (**14**), useful as an anchor group on the magnetic nanoparticles (Scheme 2). To remove the excess of unbonded nitrodopamine, the yellow solid was dialyzed in Milli-Q water for 48 h and then dried (freeze-drying).

**Figure 1:**
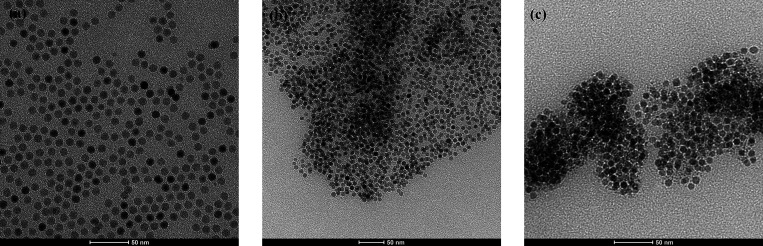
Transmission electron micrographs for (a) oleic acid-coated SPMNPs and (b and c) FeOx@PAmOx-b-PiPrOx SPMNPs grafted with nitrodopamine-functionalized block copolymers. Only the cores have sufficient contrast to be visible, but the impact of the shell grafting is observed by the separated cores that were aggregated by the shells during drying.

Molecular weight analysis by gel permeation chromatography (GPC) was performed before the addition of the second monomer (21,175 Da) and at the end of the block copolymerization. For the block copolymers, the molecular weight is 49,404 Da, nearly doubled after polymerization of the second monomer, a value that confirms successful polymerization. Furthermore, the GPC analysis shows a polydispersity index of 1.1, which confirmed linear chain extension of the polymer (Table 1). Before grafting the block copolymer on the central core, we provided to remove the carbamate protecting group from the amine functionality. Specifically, trifluoroacetic acid (TFA) was added to the corresponding polymer dissolved in dichloromethane (DCM), and the final solid product was isolated, precipitating the polymer into a mixture of Hex/Et_2_O (1:1). Spherical, monodisperse, and single-crystalline iron oxide nanoparticles were synthesized using a modified heat-up method introduced by Hyeon and co-workers.^45^ Oleic acid-coated SPMNPs were obtained by thermal decomposition of iron(0)pentacarbonyl in dioctyl ether in the presence of oleic acid. The size distributions of the SPMNPs were characterized by transmission electron microscopy (TEM). Figure 1a shows TEM micrographs of 8.5 ± 0.3 nm superparamagnetic iron oxide cores.^14^ In order to ensure fast and full ligand replacement, the grafting of nitrodopamine (NDA) block copolymer to the SPMNPs cores was performed in large excess of polymers following the previously established protocol for replacement of oleic acid by NDA-functionalized polyoxazoline.^17,46^ This procedure allowed us to prepare, after purification by dialysis, high grafting density SPMNPs.

**Table 1:**
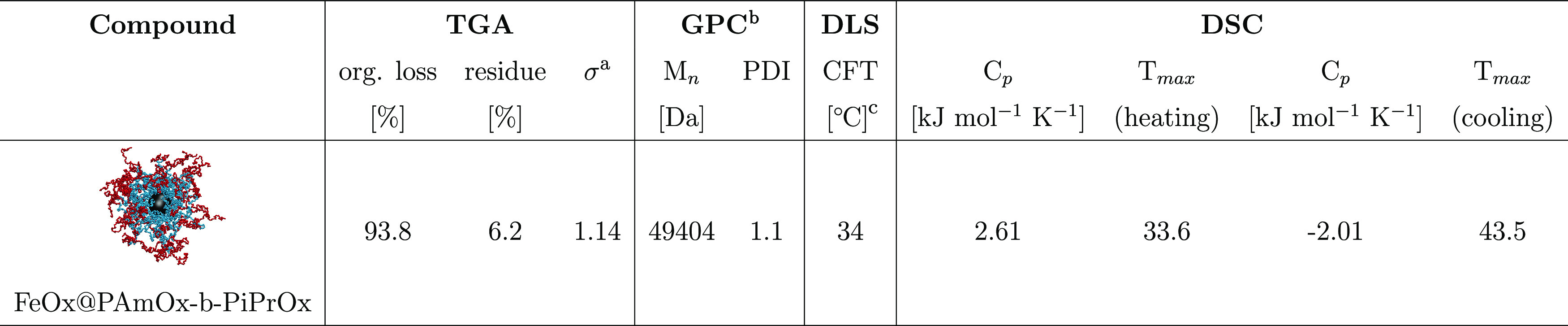
Characteristics of SPMNPs Grafted with Block Copolymer

a Grafting density.

b The measurement refers to the free polymer.

c Critical solution temperature for the poly(2-isopropyloxazoline) block measured in the free polymer.

Analysis of the final product by thermogravimetric analysis (TGA) showed grafting densities of 1.1 chains nm^–2^ for the original block copolymer FeOx@PAmOx-b-PiPrOx. TEM investigation was performed under ultrahigh vacuum (UHV) after deposition of the aqueous dispersions of nanoparticles on TEM grids. Figure 1 shows TEM pictures of: (i) ordered dispersion of dried oleic acid-coated nanoparticles, where monodispersed particle−particle distances can be easily observed (frame a); (ii) the synthesized SPMNPs, in which the aggregation between particles is due to interactions among the polymer grafted chains (frame b and c). The distance between the polymer-coated Fe-Ox nanoparticles is not monodispersed, which could be attributed to the presence of a dense polymer shell that shields core interactions but can produce limited attractive interactions in solution or during drying. Only the cores have sufficient electron density to be visible in the TEM micrographs.

### Temperature Dependence of Aggregation: Free Polymer *versus* Core–Shell Nanoparticles

**Figure 2:**
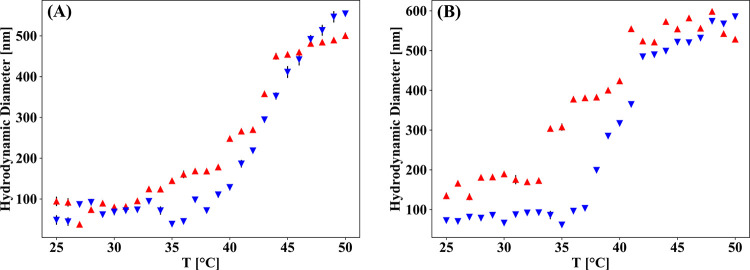
DLS heating (red triangles) and cooling (blue triangles) curves with number-weighted hydrodynamic diameter vs temperature for free block copolymer (A) in Milli-Q (1 mg mL^–1^). Size vs temperature for FeOx@PAmOx-b-PiPrOx (B), mean values, and standard deviations from three measurements.

The size, colloidal stability, and thermal responsiveness of the dispersed polymer-grafted SPMNPs were studied using dynamic light scattering (DLS). DLS was then used to further elucidate the thermal transition of the grafted block copolymer.^47^ The critical solution temperature (CST) describes the temperature above which a transition of the polymer occurs from soluble to poorly soluble in a medium, *e.g.*, water. This transition can be observed by turbidity measurements or DLS as the temperature at which aggregates form and light starts to scatter from the polymer dispersion.^48^ The CST of a polymer is deeply influenced by many parameters, such as the concentration,^49^ the end group,^50^ the monomer composition,^51^ and the type and concentrations of ions in the aqueous surrounding. Nanoparticles grafted with thermoresponsive polymers can show a much more complex behavior, with the grafted polymer undergoing multiple thermal transitions corresponding to CSTs. These appear because the local polymer concentration within a brush of high curvature varies radially from the core surface.^52,53^ These multiple transitions can be detected by differential scanning calorimetry (DSC), which directly measures the CST as the loss of hydration of the polymer.^47^ Only one transition is observed if the grafted polymer is dominated by one density regime. Thus, the polymer-coated nanoparticle dispersion can lose its colloidal stability as the result of the CST transition of part or the whole of the polymer shell. The temperature at which this flocculation of the nanoparticles occurs is called the critical flocculation temperature (CFT). It can be observed by DLS as a sudden change in scattering or cluster size.^47^ In very stable polymer-coated or hydrogel particle dispersions, the CST of the polymer can even be associated with an initial slight decrease in average particle size observed by DLS before aggregation dominates at a second (higher) CFT, yielding an increase in average size. We investigated the colloidal stability of the block copolymer-grafted SPMNPs by DLS in the temperature range of 25–50 °C at a concentration of 1 mg mL^–1^ in Milli-Q water. Further, we compared the thermally induced aggregation of the polyoxazoline block copolymers with their nanoparticle-grafted analogs. The free-coil block copolymer samples show aggregation upon heating with a clear step-like increase in size from ∼100 nm in diameter to ∼500 for NDA-PAmOx-b-PiPrOx (Figure 2A, red triangles).

These transitions can be assigned to the PiPrOx block, which is expected to have a CST in this temperature range. A CST of 38 °C was found for the block copolymer. The initial polymer dispersion is reobtained upon cooling almost without hysteresis (blue triangles) due to the rehydration of the polymer, as shown in Figure 2A. The large average size of the polymer below the CST indicates that the free polymer form aggregates that could be micellar structures, although the size measured by DLS is larger than what is expected for spherical micelles, but clearly there is some affinity between the free polymer coils in Milli-Q water. Since PiPrOx is known to be soluble as individual coils up to higher volume fractions than used here,^54^ the attractive interaction must be an effect of the introduction of the novel PAmOx blocks. Just like the free polymer coils, the SPMNPs show the formation of small clusters of maximally a few nanoparticles also below the CST, with average hydrodynamic diameters of 100–130 nm for FeOx@PAmOx-b-PiPrOx (Figure 2B). The clustering is in agreement with the weakly attractive behavior observed also in TEM images of dried samples (Figure 1b,c). Notably, the cluster size is small and, although it increases above the CFT, the size remains in the sub-micron level/range, indicating a high overall colloidal stability of the core–shell nanoparticle dispersion. The DLS plots displayed the CFT for the SPMNPs to be lower than the CST of the micelle-forming free polymer for FeOx@PAmOx-b-PiPrOx, according to the common behavior of polymer brushes. The CFT of the SPMNPs is observed at ∼34 °C compared to a CST of ∼38 °C for the NDA-PAmOx-b-PiPrOx free polymer.

The polymer shows a sharper cooling CFT transition, which is an even more pronounced difference for the SPMNP.

In conclusion, the T-cycled changes measured through DLS, especially in the hydrodynamic diameter, highlight the strong impact that grafting a thermoresponsive polymer has on the transition temperature of the coated SPMNPs. Significantly, the thermal responsive behavior is maintained after grafting and with the presence of an ionizable side chain due to the inner position of that block of the entire SPMNPs, which will enable magnetic extraction from colloidal stable SPMNP dispersions.^17^

### Investigation of the Transition Enthalpy by DSC

**Figure 3:**
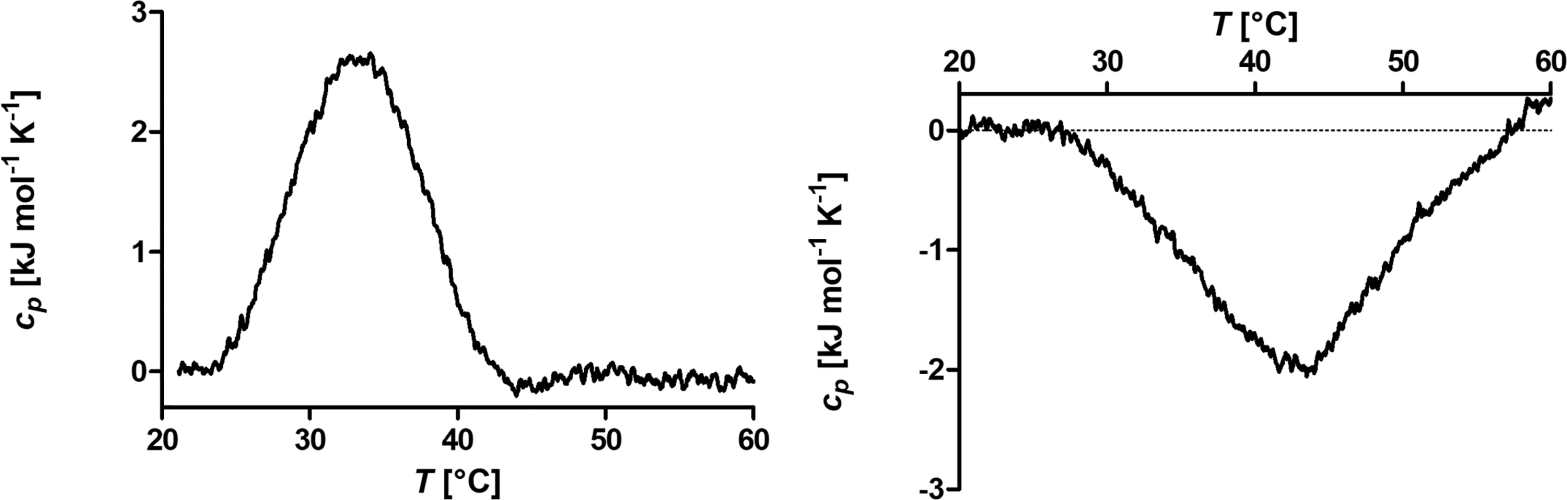
DSC heating (sx) and cooling (dx) curves for FeOx@PAmOx-b-PiPrOx in Milli-Q (1 mg mL^–1^).

The dispersions in water of SPMNPs were investigated by DSC to get further insights into the thermal solubility transition of the PiPrOx in the shell in the temperature range of 20–60 °C (Figure 3). The same mass concentration of 1 mg mL^–1^ was used as in the DLS measurement. For all samples, a single endothermic peak is observed. Given that the PiPrOx block is located in the less dense outer part of the shell, it is not surprising that a single, broad transition peak is observed. This transition is driven by the entropic favoring of bulk water above the CST, which leads to the breaking of hydrogen bonds between the PiPrOx blocks and water molecules detected in DSC. The transition temperatures determined as the peak in the specific heat capacity of the PiPrOx block after grafting on the SPMNPs is 33 °C for FeOx@PAmOx-b-PiPrOx during heating and 43 °C during cooling (Figure 3). These values are in surprisingly good agreement with the CFT values for the colloidal transition determined by DLS. That a single homogeneous transition is observed in DSC leads to a strong correlation between the observed CFT and DSC transitions, despite the differences in experimental setup, heating and cooling rates.

### Investigation of the Adsorption Properties by ITC

**Figure 4:**
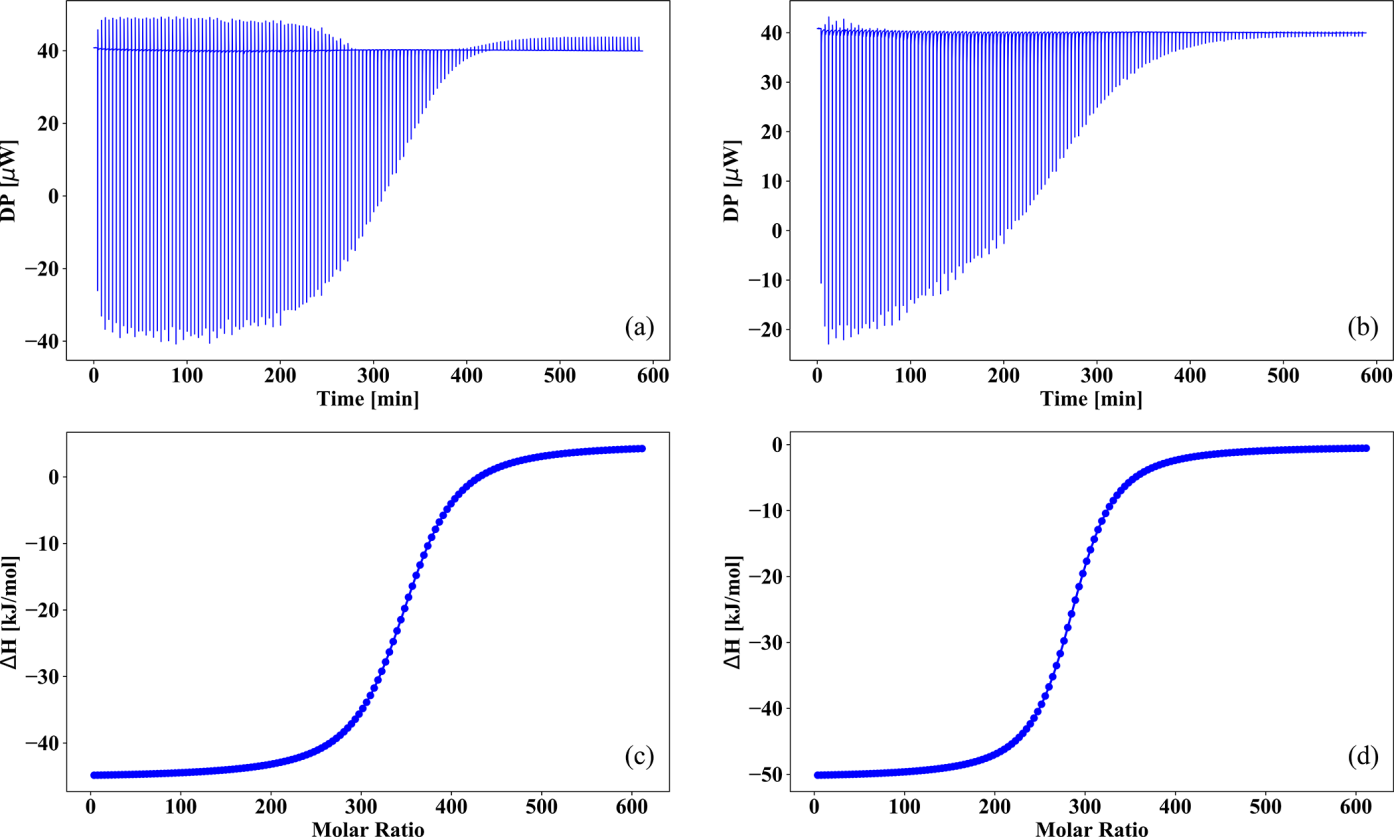
Binding thermogram and isotherm for SPMNPs–mercury(II) (panels (a) and (c)) and for SPMNPs–chromium(IV) (panels (b) and (d)); the interactions reported in both top and bottom panels are an average performed over two different measurements.

Isothermal titration calorimetry (ITC) is a reliable technique to sensitively investigate macromolecule and nanoparticle interactions with other molecules and ions in solution.^55−57^Figure 4 shows the ITC measurements of the adsorption process between the synthesized SPMNPs and two heavy metal ions (Cr(IV) and Hg(II)) in water solution. The usual procedure is to titrate a solution of compound A (50 nM aqueous solution of SPMNPs) placed in the calorimetric cell by aliquots of a solution of compound B (50 mM aqueous solution of heavy metal salt) placed in an automated syringe. The exchanged heat is measured by calculating the differential power used to maintain the reference and sample cells in thermal equilibrium for each injection and reveals quantitative information about the interaction between A and B as shown in the top row graphs of each panel of Figure 4. The enthalpy for each injection is calculated by integrating the heat capacity for the corresponding injection after subtracting baseline effects such as the heat of dilution. The latter is observed as residual injection peaks after the interaction has been saturated by multiple injections. We use the fitted offset method to remove this contribution from the data by subtracting the average enthalpy of the last injections from each data point. The stoichiometric parameter, *n*, of the binding process is defined as the molar ratio at the inflection mid-point of the enthalpy curve, while the dissociation constant *K*_D_ is defined as the slope of the isotherm curve at this point. The entropy of the interaction Δ*S* can be derived through the Gibbs equation, from the direct measurement of the enthalpy changes Δ*H*:
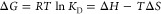
1where Δ*G* is the Gibbs free energy, *R* is the gas constant, and *T* is the experimental temperature; *n* and *K*_D_ can be determined at almost any stoichiometry between A and B within the sensitivity of the measurement.

All thermodynamic quantities attributable to the binding process were extracted from the thermograms produced from each titration after the subtraction of the heat of dilution effects. Finally, we used a differential binding model (DBM) considering just one set of identical binding sites.^58^ The number of SPMNPs per the cell is chosen to render the concentration of binding monomers, belonging to the SPMNPs, of the same order of magnitude the concentration of injected ions (mM). As shown in Figure 4 and Table 2, the DBM model fitted the experimental *K*_D_ for both chromium and mercury, which are found to be 32.3 and 41.3 μM, respectively. The similarity between the two *K*_D_ demonstrates an efficient binding between SPMNPs and the corresponding heavy metal ions. In the ITC thermogram, noteworthy is a negative heat peak, which evinces that the reaction is an exothermic process. The negative Gibbs energy values in the binding processes (Δ*G*_Hg(II)_ = −25.1 kJ/mol and Δ*G*_Cr(IV)_ = −25.8 kJ/mol) indicate that the binding reaction is thermodynamically favorable. Furthermore, negative Δ*H* values (Δ*H*_Hg(II)_ = −51.1 kJ/mol and Δ*H*_Cr(IV)_ = −50.7 kJ/mol) prove that the reaction is an exothermic process, while the additional negative Δ*S* values (−*T*Δ*S*_Hg(II)_ = 24.9 kJ/mol and −*T*Δ*S*_Cr(IV)_ = 26.0 kJ/mol) in these system indicate that weak short-range interactions play an important role during the adsorption process. Finally, the obtained Δ*G* values suggest a structural disorder decreasing in the final solution as well as the formation of stable SPMNPs–heavy metal complex. The similarity in the thermodynamic values obtained for the adsorption of the two different metallic ions suggests a strong analogy for the two species. The number of binding sites found is again similar for the two heavy metals and greater than one, *n* ≈ 3.

**Table 2:** Thermodynamic Parameters from the ITC Experiment Calculated per Monomer of AmOx in the SPMNP Dispersion

sample	*n* [sites]	*K*_D_ [μM]	Δ*H* [kJ/mol]	Δ*G* [kJ/mol]	–*T*Δ*S* [kJ/mol]
chromium [Cr(IV)]	2.85	32.3 ± 3.7	–50.7 ± 0.61	–25.1	26.0
mercury [Hg(II)]	3.43	41.3 ± 2.6	–51.1 ± 0.41	–25.8	24.9

In the following section, we conducted a theoretical and computational investigation that, exploiting the importance of the short-range interaction in the adsorption process, analyzes the whole process and the way in which ions distribute within the SPMNPs. The experimental finding that *n*>1 rather suggests that ions in solution might cluster inside the brush or on the core of the polymeric macromolecule.

### Theoretical Approach to the Adsorption Process

We performed a simplified theoretical analysis to analyze the various phases of the adsorption process; by neglecting all chemical details, we studied how the size ratio between the cargo and the adsorbing macromolecule as well as the interaction between the monomers of the macromolecule and the cargo affect adsorption.

Moreover, to assess the experimental ITC results, the maximum loading per particle is investigated.

The experimental analyzes performed on the SPMNPs showed that the average size of the coated nanoparticles is way bigger than the core size; this renders each coated SPMN, for practical purposes, an effective star polymer. We will thus simplify the theoretical representation of the system, by representing the coated SPMNPs as star polymers made by *f* arms of *n*_s_ monomers each tethered on a central core of negligible size.

The radius of gyration *R*_s_ of a free non-adsorbing star can be expressed as a function of the number of arms *f*, constituting the macromolecular assembly, and the number *n*_s_ of monomers belonging to each arm:^59^
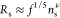
2where *ν* is the Flory exponent, which indicates the interaction of the monomers with the solvent. As all of the experimental part of this work has been performed in good solvent, and the solvent quality has been left unchanged during the experimental ITC adsorption analysis, we will set *ν* to be the good solvent Flory exponent *ν* = 0.588.

In the theoretical and computational approach, ions will be described in an oversimplified way as target particles (TP) of average radius *r*_TP_ = *R*_s_/*q*.

The theoretical investigation *q* is chosen so that size of the target particles is of the same order of magnitude of the size of monomers of the star, *e.g.*, in this representation *q* ∈ [40,80]. This should be compatible with the experimental conditions, where each monomer is of the order of magnitude of a few angstroms, as well as the hydrated metallic ions considered.

In the computational representation, *q* = 60 corresponds to the case in which the TP has a diameter that is comparable to the monomer diameter size σ, and *q* = 40 corresponds to *r*_TP_ = 0.78σ; thus, the radius of the TP is 1.56 times the radius of the monomer, and *q* = 80 to *r*_TP_ = 0.39σ; thus, the radius of the TP is about 0.78 times the radius of the monomer.

All experiments are performed in good solvent conditions, and intermolecular interactions within the stars are purely repulsive; no attraction is therefore present between the monomers of the star. Therefore, in the theoretical representation, all of the star monomers interact with each other through an effective entropic repulsion. To mimic the experimental attraction between the ions and the inner monomers of the stars, TPs are designed to interact with the monomers belonging to the core of the star through an enthalpic attractive interaction ε, which is tuned throughout the theoretical and computational investigation. As the heavy metal ions, in the experimental setup, are immersed in a sea of counterions, the effective interaction acting between the metal ions and the monomers of the star is a screened electrostatic interaction that can be considered, for simplicity, as a relatively short-range interaction.^60−62^ Such a choice allows for simplification in the theoretical representation of the system, as explained in detail in the section Materials and Methods.

Both enthalpic and entropic terms can be modulated by tuning the size ratio *q* and the interaction strength ε.

We performed molecular dynamics (MD) simulations, where we represented the simplified version of the experimental realization as a binary mixture made by a star polymer immersed in a sea of target TPs.

Experimental stars are made by *f* = 300 arms of *n*_s_ = 200 monomers each; therefore, they are in the so-called scaling regime, where all properties of each molecule do not depend on the particular microscopic details but on the average size of the macromolecule. For this reason, in order to investigate the adsorption properties of the system, it is sufficient to analyze star polymers that are in scaling, *i.e.*, *f* ≥ 30 and *n*_s_ ≥ 50.

In the here reported computational analysis, star polymers are made by *f* = 40 arms of *n*_s_ = 200 monomers each. To mimic the experimental realization, each arm as a dual interaction with the TPs, the first *n*_h_ = 100 monomers interact with the TPs in solution by means of an attractive Lennard-Jones (LJ) potential with varying interaction strength ε, while the remaining *n*_t_ = 100 interact with a purely repulsive LJ potential (see the Materials and Methods). The effective interactions between elements of the same species, *i.e.*, between TPs and between all monomers belonging to the macromolecules, are a purely repulsive LJ potential; thus, no attraction is present between elements of the same species, and only excluded volume is taken into account.

Within each diblock copolymer star, adsorption takes place in the inner shell, *i.e.*, in a sub-region of the star of radius of gyration:
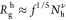
3TPs are considered to be adsorbed if their average distance *d* from the central monomer is *d* ≤ *R*_g_^h^.

Once the adsorption region has been defined, we can compute the adsorption probability *P*(*n*) as the probability that a number *n* of TPs is at a distance *d* ≤ *R*_g_^h^(*N*_ads_) from the anchor point of the star.

We can then compute the mean number of adsorbed TPs per star as
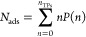
4where *n*_TPs_ is the total number of TPs considered. In the cases we analyzed, we considered a total number *n*_TPs_ = 3000. Such a number was chosen computationally as an overestimation of the maximum number that the most adsorbing system was able to load.

The adsorption process induces a shrinkage of the inner part of the molecule; TPs, being attractive to the inner monomers of the star, induce an effective attraction between the latter, thus rendering the radius of gyration an adsorption dependent quantity *R*_g_^h^(*N*_ads_).

**Figure 5:**
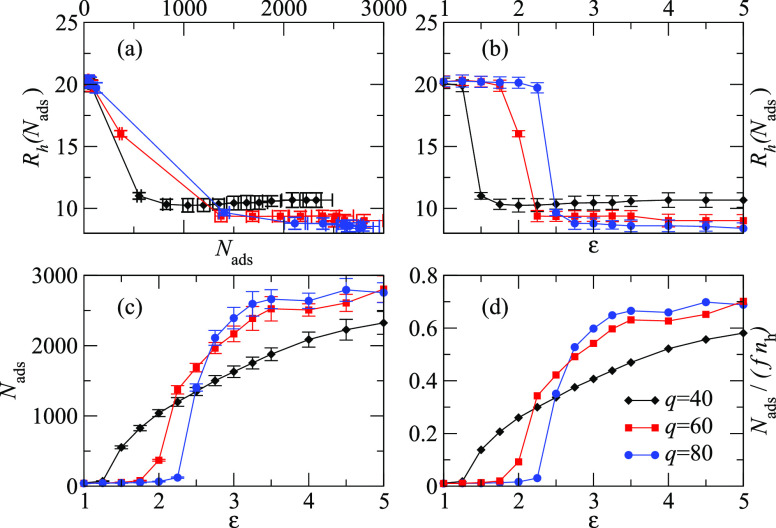
The four panels report data gathered for three different size ratios *q* = 40, *q* = 60, and *q* = 80 for ε ∈ [1,5]. (a) Radius of gyration *R*_h_(*N*_ads_) as a function of *N*_ads_; (b) the radius of gyration *R*_h_(*N*_ads_) as a function of the monomer–TPs interaction ε; (c) mean number of adsorbed TPs *N*_ads_ as defined in eq 4 plotted as a function ε; (d) the fraction *N*_ads_/(*fn*_h_) of adsorbed TPs with respect to the total number of interacting monomers belonging to the adsorbing macromolecule as a function of ε.

**Figure 6:**
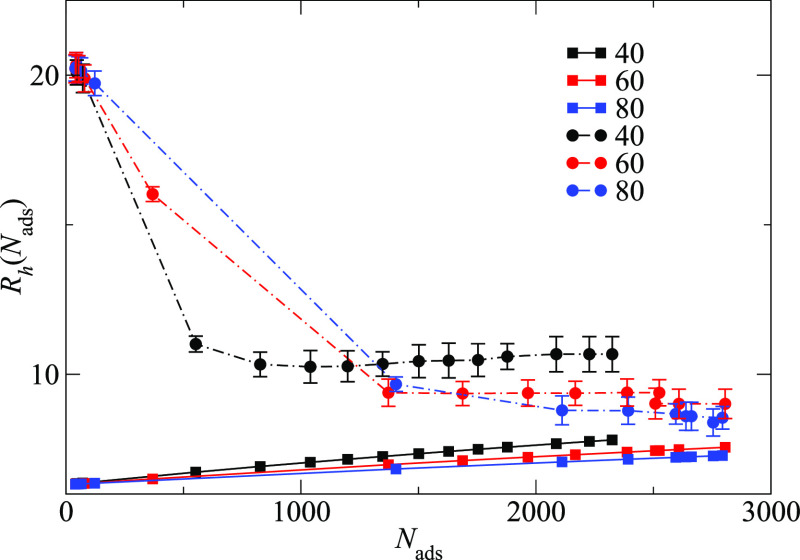
Raw comparison between the values obtained for *R*_g_^h^(*N*_ads_) as a function of *N*_ads_ for the three values of *q* analyzed (*q* = 40,60, and 80) (circles) and the radius of gyration of a collapsed star made by *f* arms of *m*_tot_ = *N*_ads_/*f* + *n*_h_ monomers each (squares). The adsorbed TPs were considered, very roughly, as a sort of “fictitious monomers”, of size *r*_TPs_(*q*)/*r*_mon_. The qualitative agreement for the trends obtained for the various *q*s is shown.

The more the adsorbed TPs, the more *R*_g_^h^(*N*_ads_) asymptotically tends to the radius of gyration of a collapsed star. We show such a phenomenon in both panel (a) of Figure 5, where *R*_g_^h^(*N*_ads_) is plotted against *N*_ads_, and Figure 6. In the latter figure, we present, only for qualitatively purposes, the comparison between the computed radii *R*_g_^h^(*N*_ads_) and the radius that a collapsed star made by *f* arms, each made of a number of monomers *n*_mon_ = *N*_ads_/*f* + *n*_h_ would have. The size of the *N*_ads_/*f* monomers is taken into account by dividing each *q*-dependent *r*_TP_ by the size of the monomer of the star: *r*_TP_(*q*)/(0.5σ). The asymptotic trends qualitatively support the statement that adsorption might be associated to an effective worsening in solvent quality for the inner monomers of the adsorbed macromolecule; the TPs particles act as a sort of effective interaction between the monomers of the star. As the adsorbed TP occupy a specific volume, their volume must be taken into account; the latter is here done in a very raw way, by adding the fraction of adsorbed TP per arm to the total number of monomers per arm of the original star, while considering their size in the computation of the radius of gyration.

As a consequence of the just described phenomenon, as the number of adsorbed TPs *N*_ads_ grows with an increase in ε, the radius of the inner region of the star shrinks as a function of an increase of the interaction ε between the monomers of the star and the TPs. This can be seen in panel (b) of Figure 5.

The dependence of *N*_ads_ on ε is shown in panel (c) of Figure 5; it clearly appears that smaller TPs require a higher ε to be adsorbed, while bigger TPs can be adsorbed even for smaller values of ε.

We can at last estimate the fraction of adsorbed TPs per total adsorbing molecules as *N*_ads_/(*fn*_h_) shown in panel (d) of Figure 5. Within the simple approach proposed in this section, we obtain a loading of about 62% of the adsorbing monomers of the macromolecule, a result that presents a qualitative agreement with what is observed experimentally, being the loading of the same order of magnitude within the two approaches.

**Figure 7:**
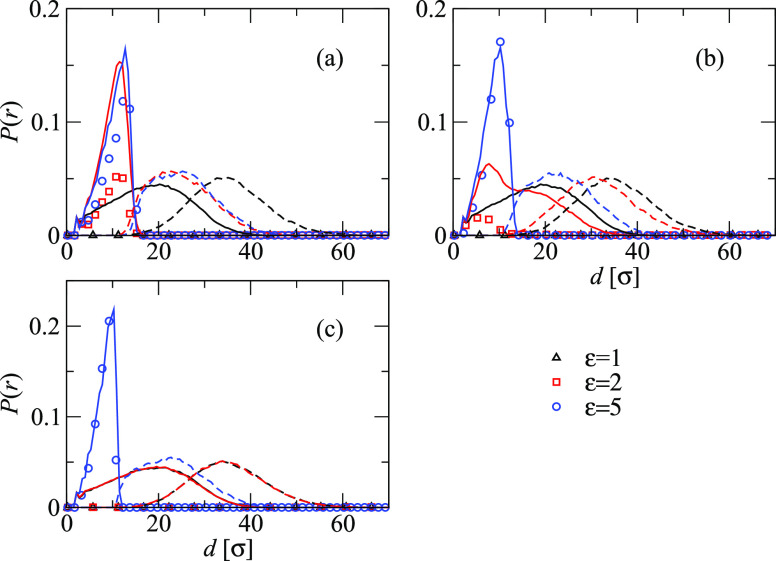
Distributions of the *h* monomers (continuous lines) and the *t* monomers of the stars (dashed lines) and the TPs (symbols) plotted as a function of the distance *d* from the central monomer of the star polymer for three different values of ε = 1 (black), ε = 2 (red), and ε = 5 (blue). Panel (a) reports the results for *q* = 40, panel (b) the distributions for *q* = 60, and panel (c) the distributions for *q* = 80.

It is also interesting to analyze how TPs distribute within the adsorbent. The shrinkage of the inner core of the SPMNPs during adsorption that we just discussed and analyzed might arise for different reasons: it could either be re-conducted to a depletion phenomenon, where all TPs would dispose around the inner core and compressed it, or, conversely, nanoparticles would distribute within the inner core of the adsorbent star, inducing an effective interaction between the monomers. Figure 7 reports the distributions of the *h* and the *t* monomers and the TPs as a function of the distance from the central monomer of the star. As soon as adsorption takes place, the TPs equally distribute in the macromolecule; we are thus seeing an adsorption process where no depletion takes place. The shrinkage of the core is then related to the “effective change in solvent quality” felt by the inner monomers of the star. Moreover, the change of size of the star, and experimentally thereby by the SPMNPs, can be an indirect measure of the number of adsorbed TPs.

## Conclusions

We successfully synthesized a versatile block copolymer composed by (i) an outer thermal responsive tail-block in aqueous solution to control the colloidal stability and (ii) an inner head-block made of monomers with an ionizable side chain (*i.e.*, amino group) that could be used to chelate predetermined compounds in solution, similar to the ethylenediaminetetraacetic acid (EDTA) behavior. These block copolymers demonstrated micellar assembly and reversible thermoresponsive aggregation with negligible hysteresis due to the CST transition of the PiPrOx block. We successfully grafted the block copolymers at a high grafting density to monodisperse iron oxide nanoparticles. The resulting core–shell nanoparticles displayed the ability to form clusters. The thermoresponsivity of the outer shell of the SPMNPs allow for thermal control of their average cluster size, as we have previously demonstrated^17,46^ and shown in Figure 2B. Thus, our design could be used for similar tuning of the aggregation by thermal actuation and magnetic extraction of the nanoparticle dispersions after absorbing heavy metal ions.^17^ The thermoresponsivity of the outer shell of the SPMNPs might allow for a thermal control of their average; this might lead to the possibility of a thermal tuning of the aggregation of the nanoparticles in solution. The FeOx@PAmOx-b-PiPrOx showed a thermoresponsive colloidal stability similar to previously studied nanoparticles grafted with PiPrOx block copolymers. Furthermore, the adsorption investigation using ITC showed that the SPMNP power of adsorption, with an ultrahigh removal efficiency for heavy metal species, sets a new benchmark for heavy metal adsorbent materials. Additionally, we performed a simple theoretical and computational analysis aimed at exploring how adsorption takes place within the nanoparticles. In the theoretical approach, we represented heavy metal ions as target particles (TPs) interacting, through a short-range potential, with the adsorbing star-like polymeric macromolecule. Such a simplification is justified as the effective interaction between ions, screened by counterions, and monomers is known to be short-ranged. The ratio between the size of the TPs and the adsorbent star polymer is set to be reminiscent of the experimental ratio between ions and macromolecules. Through the simplified analysis, we could see an adsorption of the same order of magnitude of what has been estimated experimentally. TPs distribute within the attractive part of the star polymer, inducing an effective attraction between the monomers that leads to the collapse of the inner core of the star. For the analyzed size ratios, no depletion was observed; thus, there was no phase separation between the monomers of the star polymer and the TPs. The preference of the system for an adsorption process with respect to a depletion one explains the high loading measured experimentally with ITC. Moreover, our analysis hints at quantities that can measure the macromolecule or the core–shell nanoparticle, such as the shrinkage of the core of the macromolecule or shell due to adsorption of the heavy metals, which might be used as an indirect measure of the number of adsorbed ions. This work opens the path for a combined theoretical design and experimental realization of more specialized selective adsorbers.

## Materials and Methods

### Experimental Methods

All chemicals were purchased from Sigma-Aldrich, Carl Roth, and Alfa Aesar as reported in the Supporting Information.

#### Synthesis of AmOx

##### 4-((*tert*-Butoxycarbonyl)amino)butanoic acid

A 100 mL NaOH aqueous solution (5.5 M) and γ-aminobutyric acid (0.25 mol) were placed in a 250 mL flask followed by *tert*-butoxycarbonyl anhydride (0.30 mol), and the resulting mixture was stirred overnight at ambient temperature. The reaction mixture was acidified with 1 N HCl to pH ≈ 4 and then extracted with ethyl acetate (40 mL x 3). The collected organic phase was washed with brine (30 mL x 2), dried over anhydrous MgSO_4_, filtered, and concentrated under vacuum to give the product in 88% yield (51.0 g).

##### *tert*-Butyl (4-((2-Chloroethyl)amino)-4-oxobutyl)carbamate

The first intermediate (0.20 mol), chloroethylamine hydrochloride (0.22 mol), and TBTU (0.22 mol) were dissolved in dry DCM (250 mL) sequentially. Triethylamine (0.4 mol) was added dropwise to the solution over a period of 1 h at 0 °C. The reaction mixture was allowed to warm up to room temperature and was stirred overnight before 100 mL of saturated aqueous NaHCO_3_ was added. The organic phase was washed twice with water and dried over anhydrous MgSO_4_. After removal of the solvent, the residue was distilled under reduced pressure to afford the product as a pale yellow oil (48.7 g, yield 83.2%).

##### *tert*-Butyl (2-(4,5-Dihydrooxazol-2-yl)ethyl)carbamate (AmOx)

The ring closure of *tert*-butyl (4-((2-chloroethyl)amino)-4-oxobutyl)carbamate (0.10 mol) was carried out in a saturated solution of NaOH in methanol. After stirring for 12 h at room temperature, the solvent was removed under reduced pressure. The residue was dissolved in DCM (50 mL), washed twice with water, and dried over anhydrous MgSO_4_. The desired product AmOx was obtained as a colorless solid after drying (18.2 g, yield 75.2%).

The general procedure for polymerization and grafting-to reaction were performed following the optimized protocol reported in our previous work (see the Supporting Information).^63^

#### Analytics

The complete characterization technique of SPMNPs has been performed through thermal gravimetric analysis (TGA), transmission electron microscopy (TEM), nuclear magnetic resonance (NMR), gel permeation chromatography (GPC), dynamic light scattering (DLS), differential scanning calorimetry (DSC), and isothermal titration calorimetry (ITC), as reported in the Supporting Information.

### Computational Methods

All calculations are done by means of MD simulations, performed with LAMMPS^64^ in the NVT ensemble. The simulations have been performed on a star polymer made by *f* = 40 arms, each one composed by *n*_s_ = 200 monomers, in solution with *n*_TPs_ = 3000 TPs with a volume fraction of Φ = (4/3)π(*r*_TPs_^3^*n*_TPs_ + *R*_*s*_^3^)/*V* = 0.001. We use the reduced parameters *T* = 1 (temperature) and σ = 1 (monomer diameter). The monomers within the star polymer interact with each other through a purely repulsive truncated and shifted Lennard-Jones potential (*i.e.*, good solvent conditions):
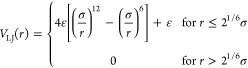
5with a cutoff set at 2^1/6^σ and ε = 1. Bonded monomers are tethered by means of a finite extensible nonlinear elastic (FENE) potential:^65^
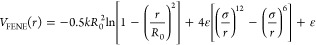
6where *k* = 30ε/σ^2^ and *R*_0_ = 1.5σ.

The first *n*_h_ = 100 monomers attached to the anchor point interact with the TPs by means of an attractive Lennard-Jones potential:
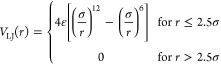
7where ε = [1,5] with *δε* = 0.25. The second half of each chain and the TPs as well as the TPs themselves interact by means of the purely repulsive Lennard-Jones potential in eq 5. TPs vary in size as a function of the ratio *q* = *R*_s_/*r*_TPs_ = 40,60,80 between the radius of gyration of the star polymer and the radius of a single TP. Simulations are performed for at least 10^8^ MD steps.

## Supporting Information Available

General synthetic procedure, ^1^H NMR spectra and GPC of free block copolymers, and TGA curves of block copolymer-modified SPION (PDF)
