# Supplementary material for: Theoretical and Experimental Design of Heavy Metal-Mopping Magnetic Nanoparticles
Source: ACS Appl Mater Interfaces. 2021 Jan 3;13(1):1386–97. doi: 10.1021/acsami.0c17759 (PMC8021223; doi:10.1021/acsami.0c17759)
Supplement: Supplementary file 1 — am0c17759_si_001.pdf [file am0c17759_si_001.pdf]

Theoretical and experimental design of heavy metal-mopping magnetic nanoparticles

Elia Roma,[a] Pietro Corsi,[a] Max Willinger,[b] Nikolaus Simon Leitner,[b] Ronald Zirbs,[b] Erik Reimhult,[b]* Barbara Capone,[a]* and Tecla Gasperi[a]*

[a]*Dipartimento di Scienze, Università degli Studi Roma Tre, Via della Vasca Navale 84, 00146, Roma, Italy*

[b]*Department of Material Sciences and Process Engineering, University of Natural Resources and Life Sciences, Vienna, Peter-Jordan-Strasse 82, A-1190 Vienna, Austria*

E-mail: erik.reimhult@boku.ac.at; barbara.capone@uniroma3.it; tecla.gasperi@uniroma3.it

Supporting Information

Contents:

1) General data

2) General procedure for the synthesis of monomers and polymers

3) Gel permeation chromatography spectra

4) TGA curves of MNPs grafted with block copolymer

5) ^{1}H NMR spectra of monomers and block copolymers

1) General data

^1H NMR spectra of polymers were measured on a BRUKER AV III 600 spectrometer. Chemical shifts were recorded in ppm and referenced to residual protonated solvent ($CDCl_3$: 7.26 ppm (1H)). Polyoxazoline molecular weights were measured by gel permeation chromatography (GPC) on an adapted Dionex HPLC utilized with a P680 HPLC pump, an ASI-100 autosampler, and an STH585 column oven. The GPC setup consists of three MZ Gel SDPlus columns (a precolumn followed by two columns with separation ranges of 10−2000 kDa and 1−40 kDa, respectively). As detector, a Knauer Smartline RI Detector 2300 was applied. As eluent, DMF with LiBr (0.05 M) was used. Samples with a concentration of 3 mg mL^{-1} were injected and measured at 60 °C with a flow rate of 0.8 mL min^{-1}. Chromeleon 6.80 with the extension pack V02 was used for analysis. Narrow dispersed polystyrene standards of 1.5−651 kg mol^{-1} were used for external calibration. Transmission electron micrographs (TEM) were recorded on a FEI Tecnai G2 with 160 kV acceleration voltage on carbon-coated grids. Thermal gravimetric analysis (TGA) of the core−shell nanoparticles were performed on a Mettler Toledo TGA/DSC with 80 mL min^{-1} synthetic air as reactive gas, 20 mL min^{-1} nitrogen as protective gas, and a heating rate of 10 K min^{-1} from 25 to 650 °C. Mass loss from 200 to 500 °C was assigned to the polyoxazoline-NDA shell, and residual mass was assigned to the inorganic core. Mass loss up to 200 °C is due to moisture or solvent residues. The grafting density was calculated from the weight fractions by TGA, the molecular weight of the block copolymer by GPC, the average iron oxide core surface determined by TEM, and a core density of 5.18 g cm^{-3}. Dynamic light scattering (DLS) measurements (hydrodynamic diameter, scattering intensity, and temperature cycling experiments) were conducted on a Malvern Zetasizer Nano-ZS. Mean values and standard deviation (number-weighted diameters) were calculated from three runs, fitting the data with CONTIN algorithm. Samples were dissolved in Milli-Q water at a concentration of 1 mg mL^{-1} and filtered with regenerated cellulose (RC) filter (0.45 µm). DSC measurements on NP dispersions in Milli-Q water (400 µL, 1 mg mL^{-1}) were performed using a MicroCal* VP-capillary DSC system at a heating rate of 1 °C min^{-1}. Data processing was done using the MicroCal VP-Capillary DSC Automated data analysis for Origin software. Enthalpy calculations are based on the mass fraction of polyoxazoline block copolymer in the material, as determined by TGA, the molecular weight of polyoxazoline block copolymer as determined by GPC, and the copolymer composition by NMR. ITC measurements were performed using Microcal PEAQ-ITC Automated (Malvern Panalytical) at 25 °C. The cell was filled with the SPMNPs dispersion. Each sample was freshly dissolved, filtered and degassed, and used on the same day. During the measurement, 2.5 µL of titrant was added every 0.25 min to the measuring cell. The first injection of 0.5 µL is customarily removed from the analysis to avoid artifacts. The data processing was done using the MicroCal PEAQ-ITC analysis software. We applied the fitting model "single set of identical sites" to best reflect the nature of interaction with a dense and uniform polymer brush shell. Baseline correction was generally done using the fitted offset routine, which estimates and subtracts the heat of dilution from the average of the converging injection enthalpies at the end of the titration curve.

Chemicals: *N,N*-diisopropylethylamine (DIPEA, 99.5 %) was dried over calcium hydride and distilled at reduced pressure. Methyl tosylate (MeTos, 98 %) was purified by distillation. *N,N*-Dimethylacetamide (DMA, anhydrous, 99.8 %) was purchased from Alfa Aesar. COMU: (1-cyano-2-ethoxy-2-oxoethylidenaminooxy) dimethylamino-morpholino-carbenium hexafluorophosphate, DMAP: 4-(dimethylamino) pyridine. Dialysis tubes (molecular weight cut-off (MWCO): 3.5 kDa (regenerated cellulose) and 1000 kDa (cellulose ester, Spectra/Por Float-A-Lyzer)) were purchased from Carl Roth.

2) General procedure for the synthesis of monomers and polymers

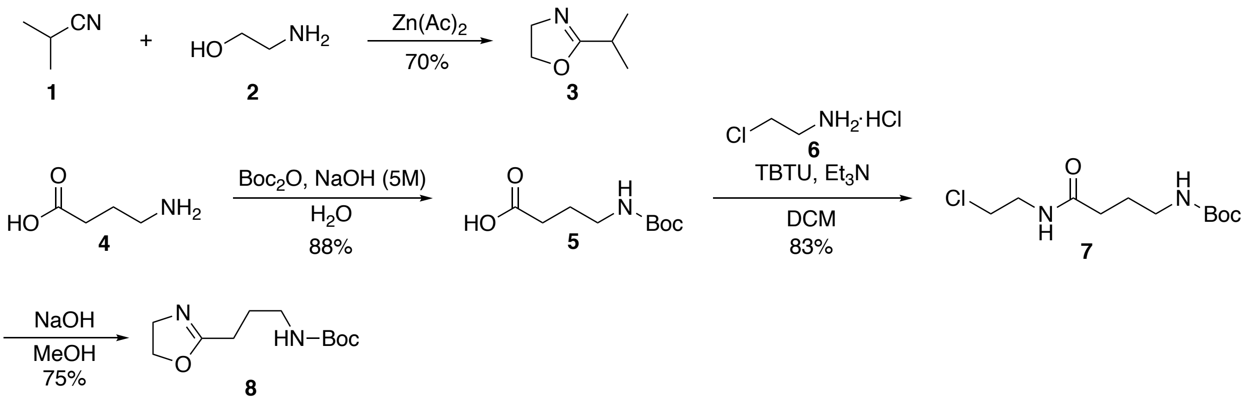

Scheme 1. Synthesis of 2-alkyl-oxazoline monomers **3** and **8**

Synthesis of IPOx (3). The monomer IPOx was synthesized via a modified Witte−Seeliger cyclocondensation[1] of isobutyronitrile with ethanolamine (1.2 equiv) and zinc acetate hydrate (0.02 equiv) as catalyst at 130 °C overnight with a flow of nitrogen passed through the reaction vessel. Technically pure iPrOx was obtained by vacuum distillation of the reaction mixture (58−60 °C at 50 mbar), followed by refluxing over calcium hydride (1−3 days) and distilling under argon. Yield: 40 ml (70 %) of a colorless liquid. ^{1}H NMR (600 MHz, $CDCl_3$): δ = 1.08 (d, *J* = 6.5 Hz, 6H), 2.47 (dq, *J* = 8.5 Hz), 3.71 (t, *J* = 9.4 Hz, 2H), 4.12 (t, *J* = 9.5 Hz, 2H).

Synthesis of AmOx. *4-((tert-butoxycarbonyl)amino)butanoic acid (**5**).* 100 mL NaOH aqueous solution (5.5 M) and γ-aminobutyrric acid (0.25 mol) were placed in a 250 mL flask, followed by *tert*butoxycarbonyl anhydride (0.30 mol), and the resulting mixture was stirred overnight at ambient temperature. The reaction mixture was acidified with 1 N HCl to pH ~4 and then extracted with ethyl acetate (40 mL x 3). The collected organic phase was washed with brine (30 mL x 2), dried over anhydrous $MgSO_4$, filtered, and concentrated under vacuum to give the product in 95% yield (51.0 g). ^{1}H NMR (600 MHz, $CDCl_3$): δ = 1.33 (s, 9H), 1.85 (p, J = 9.3 Hz, 2H), 2.36 (t, J = 9.3 Hz, 2H), 3.13 (t, J = 9.5 Hz, 2H).

*Tert-butyl (4-((2-chloroethyl)amino)-4-oxobutyl)carbamate (**7**).* The first intermediate (0.20 mol), chloroethylamine hydrochloride (0.22 mol), and TBTU (0.22 mol) were dissolved in dry DCM (250 mL) sequentially. Triethylamine (0.4 mol) was added dropwise to the solution over a period of 1 h at 0 °C. The reaction mixture was allowed to warm up to room temperature and was stirred overnight before 100 mL of saturated aqueous $NaHCO_3$ was added. The organic phase was washed twice with water and dried over anhydrous $MgSO_4$. After removal of the solvent, the residue was distilled under reduced pressure to afford the product as a pale-yellow oil (48.7 g, yield 83%). ^{1}H NMR (600 MHz, $CDCl_3$): δ = 1.33 (s, 9H), 1.85 (p, J = 9.3 Hz, 2H), 2.36 (t, J = 9.3 Hz, 2H), 2.48 – 2.64 (m, 4H), 3.13 (t, J = 9.5 Hz, 2H).

*Tert-butyl (2-(4,5-dihydrooxazol-2-yl)ethyl)carbamate (AmOx, **8**).* The ring closure of *tert*-butyl (4-((2-chloroethyl)amino)-4-oxobutyl)carbamate (0.10 mol) was carried out in a saturated solution of NaOH in methanol. After stirring for 12 h at room temperature, the solvent was removed under reduced pressure. The residue was dissolved in DCM (50 mL), washed twice with water and dried over anhydrous $MgSO_4$. The desired product **AmOx** was obtained as a white solid (18.2 g, yield 75%). ^{1}H NMR (600 MHz, $CDCl_3$): δ = 1.33 (s, 9H), 1.85 (p, J = 9.3 Hz, 2H), 2.36 (t, J = 9.3 Hz, 2H), 2.48 – 2.64 (m, 4H), 3.13 (t, J = 9.5 Hz, 2H), 3.71 (t, J = 9.4 Hz, 2H), 4.12 (t, J = 9.5 Hz, 2H).

Polymerization. Example polymerization for Me-PIPOx-b-PAmOx: polymerization reactions were performed in a Glovebox (GS Glovebox Systemtechnik GmbH) with water-level <1 ppm and oxygen-level <5 ppm. A screwcap vial equipped with a magnetic stir bar was charged with 2-isopropyloxazoline (0.42 mmol) and anhydrous DMA (2.7 mL). The flask was placed in a preheated oil bath at 100 °C. The polymerization was started by adding a solution of methyl tosylate in DMA (246 µL (stock solution: 0.1 mL of methyl tosylate in 3 mL of DMA), 0.053 mmol). After 20 h, AmOx(4.2 mmol) was added, and the polymerization was continued for another 20 h. The reaction was quenched by the addition of H_2O and stirred overnight. The polymer was precipitated by dropping the reaction solution into hexane/diethyl ether (v/v: 4/1), collected by centrifugation, and dried in vacuo. Yield: 950 mg (90%), GPC (*N,N* dimethylformamide (DMF) with LiBr (0.05 M)): 49.0 kDa, Đ = 1.1.

Carboxyl-Terminated Polyoxazoline. A solution of hydroxy-terminated block copolymer (0.047 mmol), succinic anhydride (0.45 mmol), and Et_3N (0.15 mmol) in toluene (10 mL) was refluxed for 24 h. After cooling to room temperature, the product was precipitated in hexane/diethyl ether (v/ v: 4/1). The polymer was collected via centrifugation and dried in vacuo. Yield: 700 mg (70%).

Nitrodopamine-Terminated Polyoxazoline. Carboxyl-terminated polyoxazoline (0.036 mmol), COMU (0.093 mmol), and DIPEA (50 µL) in DMF (5 mL) were reacted for 10 min at 0 °C to activate the polymer. A solution of 6-nitrodopamine hydrogensulfate

(0.12 mmol) in DMF (1 mL) was added, and the reaction solution was stirred for 3 days at room temperature. The solution was dropped into hexane/diethyl ether (v/v: 4/1) to precipitate the polymer. The yellow residue was washed with hexane, air-dried, and dissolved in Milli-Q water. The aqueous solution was dialyzed (3.5 kDa membrane cut-off size) to remove free nitrodopamine and reaction byproducts. The polymer was obtained after freeze-drying (450 mg).

Grafting-to Reaction. As prepared, oleic acid-coated MNPs (8.5 ± 0.3 nm, 100 mg) and nitrocatechol-terminated polyoxazoline (450 mg) were suspended in DMF (5 mL) and reacted under ultrasonication for 24 h.[2] The solution was dropped into hexane/diethyl ether (v/v: 4/1) to precipitate the raw product. The brown residue was washed with hexane and air-dried. The core−shell nanoparticles were purified by dialysis against Milli-Q water (MWCO: 1000 kDa) for 48 h. Polyoxazoline-modified MNPs was obtained after freeze-drying as a brown solid.

3) Gel permeation chromatography spectra

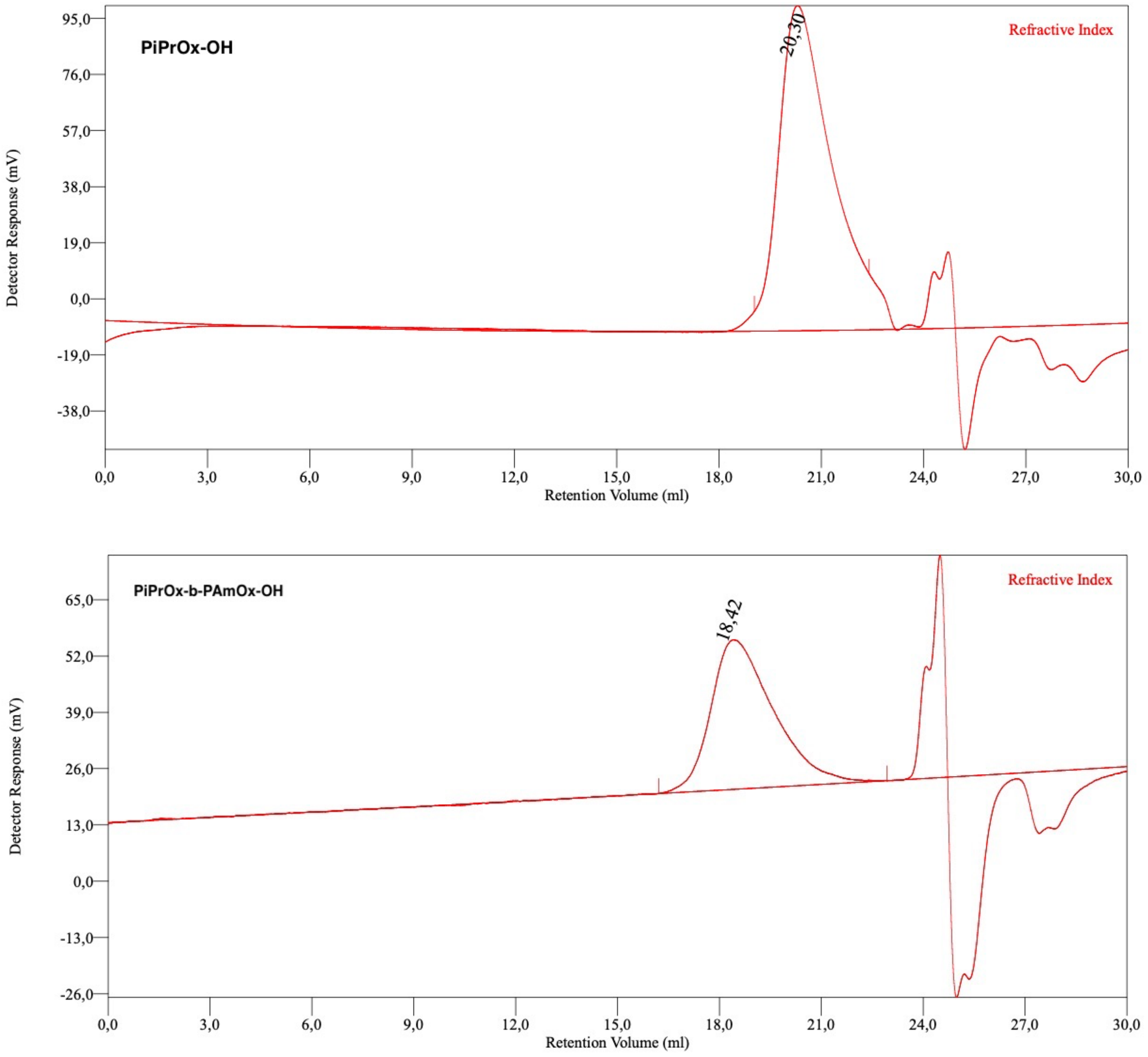

Figure S1. GPC curve for hydroxyl-terminated block copolymer PiPrOx-OH (A) and PiPrOx-b-PAmOx-OH (B), eluent: DMF/LiBr (50 mM), column temperature: 60°C, flow rate: 0.8 mL min^{-1}.

4) TGA curve of MNPs grafted with block copolymer

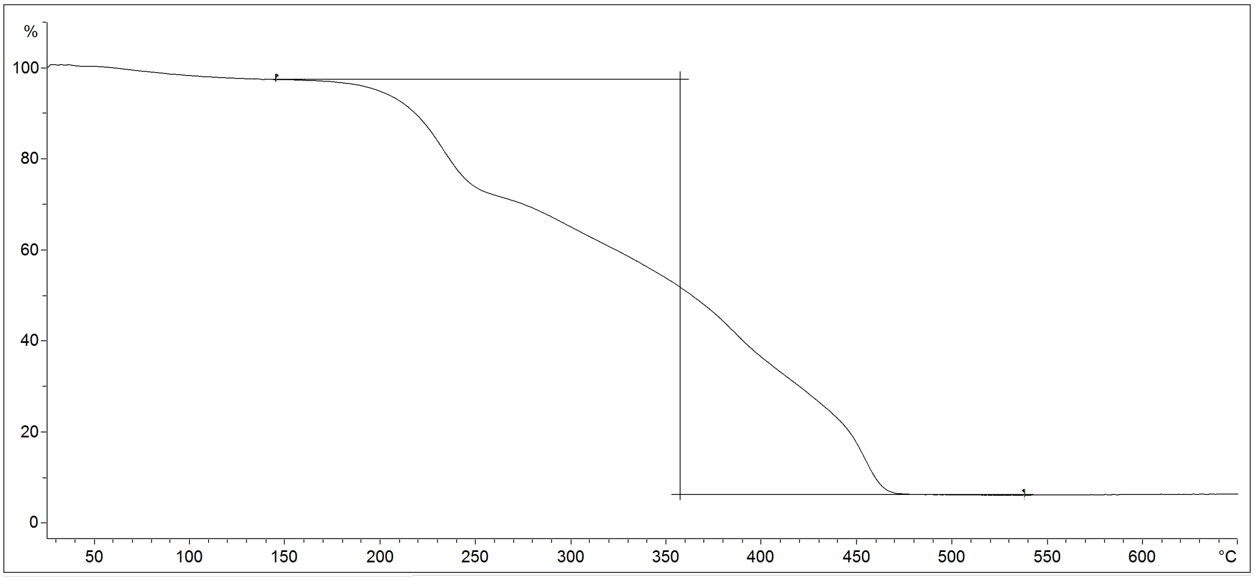

Figure S2 TGA curve for MNPs grafted with block copolymer FeOx@PAmOx-b-PIPOx (B).

5) ^{1}H NMR spectra of monomers and block copolymers

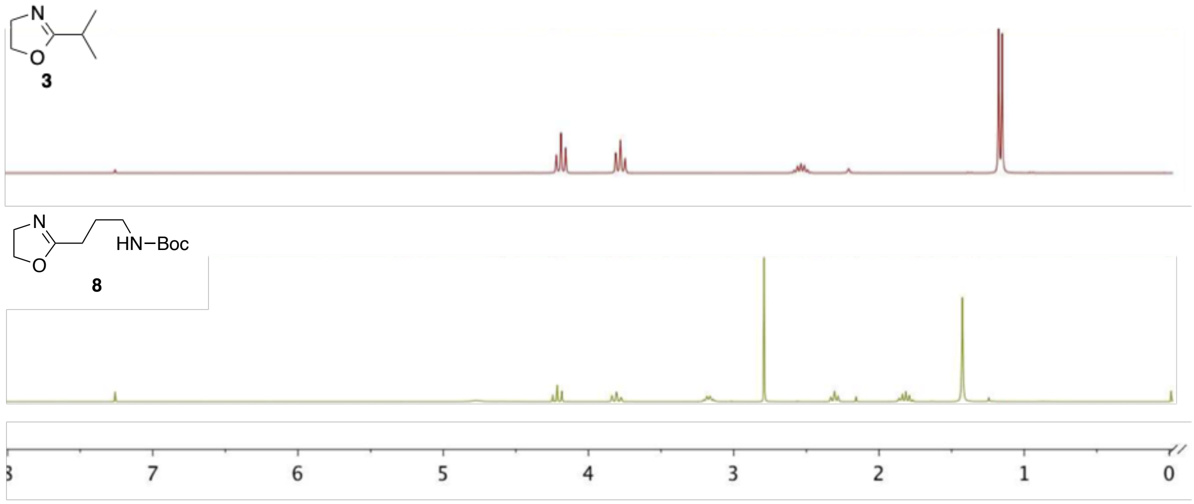

Figure S3 ^{1}H-NMR spectrum of IPOx (**3**), EstOX (**7**) and BuOX$_{NHBoc}$ (**11**) in $CDCl_3$

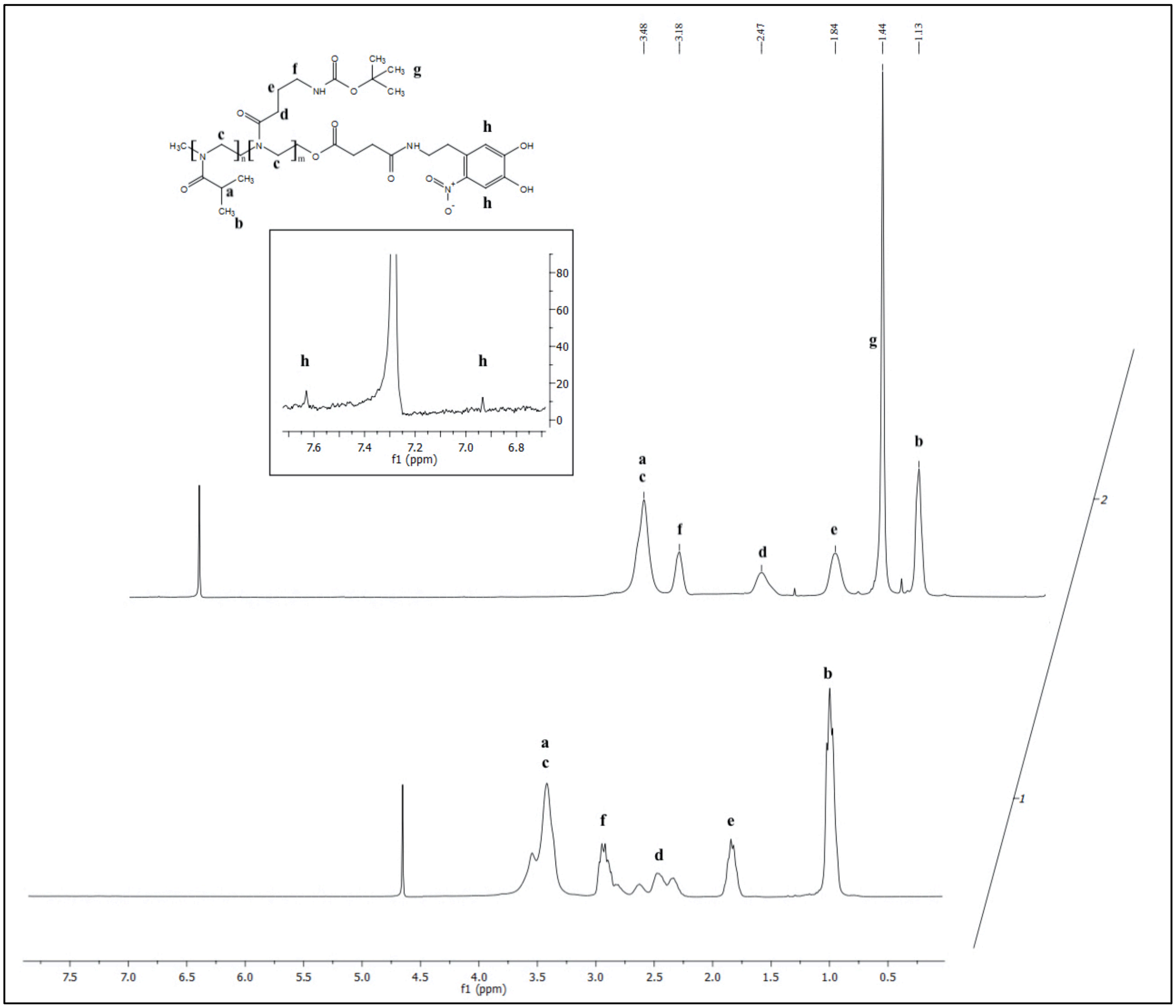

Figure S4 ^{1}H-NMR spectrum of PAmOx-b-PIPOx in $CDCl_3$ and ^{1}H-NMR spectrum of PAmOx-b-PIPOx after the protecting group cleavage in D_2O.
